# Immune-Related Adverse Events Induced by Immune Checkpoint Inhibitors and CAR-T Cell Therapy: A Comprehensive Imaging-Based Review

**DOI:** 10.3390/cancers16142585

**Published:** 2024-07-19

**Authors:** Chiara Pozzessere, Bianca Mazini, Patrick Omoumi, Mario Jreige, Leslie Noirez, Antonia Digklia, François Fasquelle, Christine Sempoux, Clarisse Dromain

**Affiliations:** 1 Department of Diagnostic and Interventional Radiology, Lausanne University Hospital (CHUV), CH-1011 Lausanne, Switzerland; 2 Department of Nuclear Medicine and Molecular Imaging, Lausanne University Hospital (CHUV), CH-1011 Lausanne, Switzerland; 3 Department of Pulmonology, Lausanne University Hospital (CHUV), CH-1011 Lausanne, Switzerland; 4 Department of Oncology, Lausanne University Hospital (CHUV), CH-1011 Lausanne, Switzerland; 5 Department of Pathology, Lausanne University Hospital (CHUV), CH-1011 Lausanne, Switzerland

**Keywords:** immunotherapy, immune checkpoint inhibitors, CAR T-cell therapy, immune-related adverse event, imaging, computed tomography, magnetic resonance imaging, positron emission tomography/computed tomography, medical oncology, toxicity

## Abstract

**Simple Summary:**

Immunotherapy has been introduced as a standard of care for several cancers, and its use is on the rise. However, the enhanced immune system frequently causes immune-related adverse events. Depending on the affected organ and tissue and the severity of the toxicity, immunotherapy is generally held, and steroids or immunosuppressive agents are introduced, impacting cancer treatment. Therefore, prompt identification of toxicity at an early stage and multidisciplinary management are mandatory. Since imaging is crucial to guide clinicians in managing immune-related adverse events, this imaging-based review presents the most frequent complications identifiable on imaging. Clues for identification and the most important differential diagnoses are discussed to enhance knowledge among imaging specialists and clinicians regarding these complications.

**Abstract:**

Immunotherapy has revolutionized oncology care, improving patient outcomes in several cancers. However, these therapies are also associated with typical immune-related adverse events due to the enhanced inflammatory and immune response. These toxicities can arise at any time during treatment but are more frequent within the first few months. Any organ and tissue can be affected, ranging from mild to life-threatening. While some manifestations are common and more often mild, such as dermatitis and colitis, others are rarer and more severe, such as myocarditis. Management depends on the severity, with treatment being held for >grade 2 toxicities. Steroids are used in more severe cases, and immunosuppressive treatment may be considered for non-responsive toxicities, along with specific organ support. A multidisciplinary approach is mandatory for prompt identification and management. The diagnosis is primarily of exclusion. It often relies on imaging features, and, when possible, cytologic and/or pathological analyses are performed for confirmation. In case of clinical suspicion, imaging is required to assess the presence, extent, and features of abnormalities and to evoke and rule out differential diagnoses. This imaging-based review illustrates the diverse system-specific toxicities associated with immune checkpoint inhibitors and chimeric antigen receptor T-cells with a multidisciplinary perspective. Clinical characteristics, imaging features, cytological and histological patterns, as well as the management approach, are presented with insights into radiological tips to distinguish these toxicities from the most important differential diagnoses and mimickers—including tumor progression, pseudoprogression, inflammation, and infection—to guide imaging and clinical specialists in the pathway of diagnosing immune-related adverse events.

## 1. Introduction

Immunotherapy refers to a type of treatment that uses the body’s immune system to fight cancer. It includes various approaches: immune checkpoint inhibitors (ICIs), chimeric antigen receptor T-cell (CAR-T cell) therapy, cancer vaccines, interferons and interleukins, and monoclonal antibodies.

ICIs, including programmed death-ligand 1 (PD-L1), programmed death-1 (PD-1), and cytotoxic T-lymphocyte antigen-4 (CTLA-4), are currently the standard of care for different histologic types of cancers. By enhancing the activity of the immune system, ICIs may induce inflammatory side effects through diverse mechanisms. These include the heightened activity of T-cells not only within tumors but also in healthy tissues. Additionally, ICIs can elevate the levels of pre-existing antibodies and boost the production of inflammatory cytokines, such as IL-17. Moreover, they have the potential to upregulate the complement inflammatory system, a cascade of proteins that promotes inflammation and cellular damage [1].

Monotherapy has demonstrated superior tolerability compared with chemotherapy. In a randomized study involving patients diagnosed with non-small cell lung carcinoma, the incidence of treatment-related adverse events was compared between patients receiving atezolizumab (anti-PD-L1) and those administered docetaxel. They found a lower occurrence of treatment-related grade 3 or 4 adverse events among atezolizumab-treated patients (90 out of 609, accounting for 15%) as opposed to those treated with docetaxel (247 out of 578 patients, accounting for 43%) [2]. Notably, the association between the effectiveness of immunotherapy and the occurrence of adverse reactions has been identified in several studies, with a better prognosis observed in patients experiencing immune-related adverse events (irAEs) [3,4,5].

IrAEs can affect any organ and system, but they tend to most frequently target skin, endocrine glands, gastrointestinal tract, liver, and lungs. Although some tissues are less frequently involved, there is an inverse correlation between their frequency and severity, except for peripheral neurological disorders. For example, colitis is a common side effect but rarely results in high-grade treatment-related adverse events. Conversely, myocarditis is uncommon but tends to be more severe. These side effects can manifest as localized inflammatory conditions in a single organ, such as dermatitis, or as systemic diseases. The adverse effects of ICIs are low-grade in most cases. High-grade events occur more frequently with anti-CTLA-4 compared to anti–PD-1/PD-L1 therapy and the combined use of CTLA-4 and PD-1 antibodies exhibits the highest incidence of any-grade and severe (grade 3 or 4) toxicities, reaching up to 95% and 55%, respectively [6,7]. Additionally, the spectrum of immune-related adverse events varies between these two classes of ICIs. Pneumonitis and thyroiditis are more prevalent in anti-PD-1 therapy, while hypophysitis and colitis are more commonly associated with anti-CTLA-4 therapy [8]. Although most irAEs arise within the initial months of treatment, they have the potential to manifest at any point throughout the course of immunotherapy even after discontinuation [9].

CAR-T cells are genetically engineered immune cells used in hematologic neoplasia and are designed to recognize specific proteins expressed in the surface of B cells or plasma cells. Adverse reactions from CAR-T cells are primarily due to the activation of the immune system and can be severe. The main cause of these reactions relies on the massive cytokine release.

As immunotherapy continues to revolutionize cancer treatment and has become the standard of care, the diagnosis and management of toxicities have become paramount. Treatment strategies often involve a combination of immunosuppression and supportive care. Close multidisciplinary collaboration between clinical specialists (oncologists, immunologists, pneumologists, endocrinologists, gastroenterologists, neurologists), diagnostic imaging specialists, and pathologists is essential to tailor interventions based on the specific characteristics of irAEs and the patient’s overall health. Early diagnosis is crucial, particularly in case of neurological and cardiac complications due to the high mortality.

This review establishes a foundational framework and showcases the radiological features and differential diagnosis of the most prevalent ICI and CAR-T cell toxicities, currently the standard of care in many cancers, with the aim of enhancing knowledge of these adverse events for deep collaboration among pathologists, oncologists, and organ specialists.

## 2. Adverse Events Related to Immune Checkpoints Inhibitor Therapy

### 2.1. Neurologic Adverse Events

Neurological toxicities are rare but severe events, accounting for 1–5%, depending on the treatment type [10]. They are classified into two different main categories, depending on the type of treatment that causes them: neurologic immune-related adverse events (irAE-Ns), secondary to immune checkpoint inhibitors (ICIs), and neurological toxicities secondary to chimeric antigen receptor cell therapies T (CAR T).

Regarding irAE-Ns, the spectrum is large, with potential affection of the entire neuroaxis: the central nervous system (CNS; encephalitis and aseptic meningitis) and the peripheral nervous system (acute and chronic demyelinating disease and myelitis, cranial nerve neuropathies, myasthenic syndromes, and myositis) [11]. However, peripheral nervous system iRAE-Ns occur twice as often as central nervous system iRAE-Ns [12].

The incidence of iRAE-Ns changes with the type of treatment: in a meta-analysis of 59 clinical trials the percentage of patients with irAE-Ns is 3.8% while on anti-CTLA-4 inhibitors, 6.1% while on anti-PD1 inhibitors, and 12.0% while on both in combination [13]. A recent pharmacovigilance study also reported some associations between the class of ICI and specific iRAE-Ns; particularly, they found that non-infectious encephalitis/myelitis are more frequent with anti-PD-1/PD-L1 than with anti-CTLA-4, while Guillain-Barre syndrome and non-infectious meningitis are less frequently reported with anti-PD-1/PD-L1 compared with anti-CTLA4 monotherapy [14].

Early diagnosis of neurological toxicities is crucial, considering the potential for rapid progression, high morbidity, and mortality. Diagnosing neurotoxicity can be particularly challenging because of the nonspecific clinical symptoms and radiological features. Therefore, an extensive workup for potential differential diagnoses, including brain/medullary magnetic resonance imaging (MRI), lumbar puncture (LP), paraneoplastic autoantibodies, electroencephalogram (EEG), and laboratories, notably to rule out infectious, vascular, toxic and metabolic causes. Moreover, in patients with brain metastasis, the radiological interpretation can be particularly challenging due to the overlap of radiological semiology between toxic lesions, tumor progression, and tumor pseudoprogression. MRI brain protocol depends on the clinical suspicion; however, it should always include T2w, FLAIR, DWI, and pre and post-contrast enhancement T1 images. Table 1 shows neurotoxicities related to the ICIs, diagnostic work-up with radiological clues, and main differential diagnoses. Guidelines for the management of neurological adverse events rely mostly on expert opinions and knowledge of autoimmune diseases.

#### 2.1.1. Ir-Encephalitis

Ir-encephalitis is the most common CNS irAE, with an incidence of 0.1–0.2% [14,16], and it has been described with ICIs [15]. Symptoms include confusion, agitation, cognitive impairment, fever, headache, seizure, and psychiatric disturbances [17].

The diagnostic workup usually includes contrast-enhanced brain MRI, LP, and EEG, with the main goal of ruling out infection and disease progression. Indeed, MRI can be normal. When pathological, T2 and fluid-attenuated inversion recovery (FLAIR) sequences play a central role in this diagnosis, showing ill-defined uni- or bilateral hyperintensities in the mesiotemporal lobes, basal ganglia, cortico-subcortical areas, and cerebellum. Contrast enhancement is variable and, if present, can be secondary to either inflammatory infiltrated or epileptogenic activity [18].

#### 2.1.2. Ir-Aseptic Meningitis

Aseptic meningitis is another rare but severe ICI complication, affecting <0.1% of patients [17]. Clinical presentation is nonspecific, including headache, neck stiffness, fever, nausea, and photophobia [17]. The diagnostic workup usually includes PL and contrast-enhanced brain MRI. Brain MRI can show leptomeningeal enhancement, leptomeningeal T2/FLAIR hyperintensities, or nerve root enhancement (8%) [17]. Although MRI may be normal in up to 46% of cases, this examination allows the ruling out of alternative diagnoses such as infectious or carcinomatous meningitis, brain metastasis, and vascular events.

#### 2.1.3. Ir-Central Nervous System Vasculitis

Even though the association between ICIs and central nervous system vasculitis has been described in several case reports in the literature [19], the precise frequency of this side effect remains unknown [15]. Arteries of the central nervous system (primary angiitis of CNS, PACNS), the peripheral nervous system, or large vessels (giant arteritis) can be affected [15,19]. Clinical presentation is not specific, including headache, altered cognitive status, and focal neurologic deficit [15]. The diagnostic workup may include noninvasive computed tomography angiography (angio-CT) or contrast MRI, LP, serum markers associated with vasculitis, and, if necessary, biopsy [11]. When large vessels are involved, vessel abnormalities include narrowing or concentric vessel wall enhancement on vessel wall imaging [11]. On the contrary, in the case of PACNS, large and medium vessels are normal. Yet, MRI is altered in more than 90% of cases, showing signs of microangiopathy and subarachnoid hemorrhage on susceptibility-weighted imaging (SWI) sequence as well as ischemic infarction of the cortical-subcortical area on diffusion-weighted and T2-weighted and FLAIR sequences [20].

#### 2.1.4. Ir-Demyelinating Disease and Myelitis

**Figure 1:**
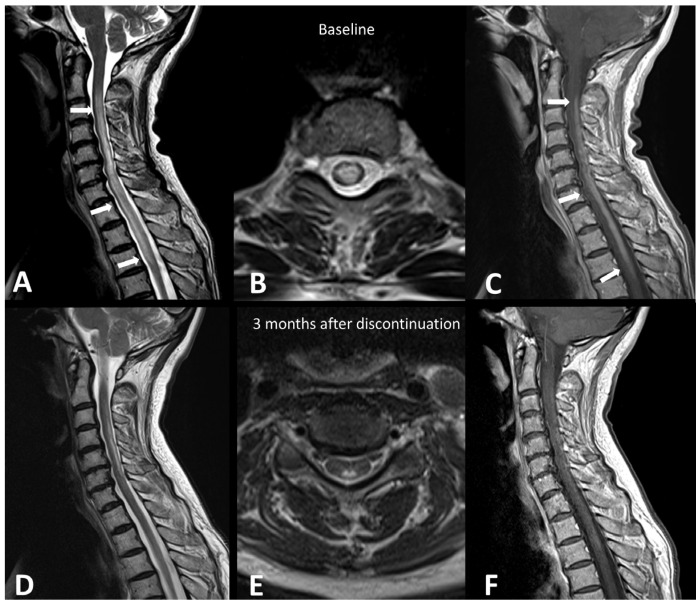
Immuno-related extensive myelitis in a patient suffering from left arm melanoma. The patient had diffuse sensitive symptoms in the forearms a few weeks after the beginning of antiPD-1 treatment. Medullary MRI revealed a T2 hypersignal on the T2 sequence involving more than three vertebrae on the sagittal plane (arrows in (**A**)) and more than 50% of the medullary diameter on the transverse plane (**B**). An extensive medullary enhancement is also seen (arrows in (**C**)). The treatment was suspended, and the patient was treated with intravenous immunoglobulin and prednisone. Medullary MRI performed 3 months after ICI discontinuation showed partial regression of T2 hyperintensities (**D**,**E**) as well as almost complete regression of the medullary enhancement (**F**).

A recent systematic literature review of case reports demonstrated that demyelinating diseases, both typical and atypical, following ICIs, are very rare but still possible [21]. Clinical presentation depends on the anatomical site of the demyelinating process. The clinical workup may include MRI of the brain, spine, and optic nerves, LP, and autoantibody evaluation. In accordance with the McDonald criteria, MRI lesions are considered demyelinating based on the presence of T2 hyperintensity and/or contrast enhancement and location in cortical, juxtacortical, periventricular, or infratentorial regions intra-cranially or in the spinal cord. In the spinal cord, hyperT2 lesions are more commonly longitudinally extensive, spanning three or more levels, and they are associated with spinal cord edema and patchy enhancement (Figure 1) [22]. MRI of the orbits can show optic nerve enhancement, typically sparing the retrobulbar and proximal segments [21].

### 2.2. Thoracic Adverse Events

Thoracic irAEs encompass checkpoint-induced pneumonitis (CIP), also known as immune-related pneumonitis (IR-P) or ICI-pneumonitis (ICI-P), sarcoidosis-like reaction, tracheitis and pleural effusion [23,24,25,26]. In Table 2, the different CT patterns of ICI lung toxicities are described along with the main clues for the identification and the main differential diagnoses.

#### 2.2.1. Ir-Pneumonitis

Ir-pneumonitis is a potentially severe adverse event associated with high morbidity and mortality [27,28]. Its development is influenced by several factors such as T-cell-mediated immune activation, disruptions in cytokine balance, autoantibody upregulation, genetic predispositions, and microbiome composition, and it may be predisposed by tobacco, preexisting lung disease (chronic obstructive pulmonary disease, asthma, interstitial lung disease), tumor type (being more frequent in non-small cell lung cancer and renal cell cancer), and previous chest radiotherapy [29,30].

The incidence of any grades of ir-pneumonitis varies depending on therapy, with combined therapies having higher rates (6.5–10%) compared to anti-PD1, anti-PDL1, and anti-CTLA-4 monotherapies (4%, 2%, and <1%, respectively) [26,27,31,32,33]. While there is no difference in the safety profile observed when used in neoadjuvant or adjuvant therapy, it has been noted that the incidence and severity of toxicities slightly increase when used as a consolidative treatment after concurrent chemoradiation [34,35]. Higher rates of any-grade and severe pneumonitis have been reported in patients receiving combination ICI with epidermal growth factor receptor tyrosine kinase inhibitors (EGFR-TKIs) [25,36]. Interestingly, in real-world settings, a higher incidence of ir-pneumonitis compared to clinical trials has been reported reaching up to 19%, possibly reflecting the less stringent eligibility criteria in clinical activity such as older age, poorer health status, and higher rates of comorbidities among patients [37].

Diagnosis is challenging due to nonspecific clinical and imaging presentations of ir-pneumonitis with differential diagnoses, including disease progression, infection, and radiation-related pneumonitis. An integrated multidisciplinary approach involving anamnestic information, imaging features, and when feasible, bronchoalveolar lavage (BAL) is crucial for diagnosis. Flexible bronchoscopy with transbronchial lung biopsies, and/or endobronchial ultrasound-guided transbronchial needle aspiration mediastinal lymph nodes can be combined with BAL in case of suspected disease progression or pseudoprogression. Nevertheless, accurately diagnosing ir-pneumonitis remains uncertain even following all the investigations, and an empiric approach guided by a multidisciplinary expert team is required.

Ir-pneumonitis can manifest within days to over a year after therapy initiation with an average onset time of around 3 months, being slightly shorter in cases of combined therapy or previous or concomitant radiotherapy [27,37]. Notably, real-world data show a later time of onset possibly due to a delayed diagnosis outside of clinical trial [38].

Clinical presentation is non-specific, ranging from mild dyspnea and/or cough to severe respiratory failure and it is graded according to the Common Terminology Criteria for Adverse Events (CTCAE) from grade 1 (asymptomatic with incidentally detected lung abnormalities at CT) to grade 4 (life-threatening).

**Figure 2:**
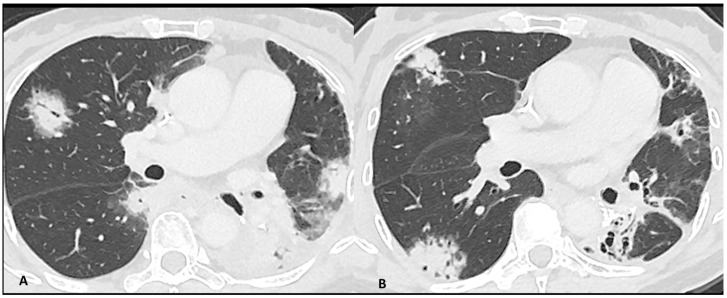
Immuno-related pneumonitis with typical organizing pneumonia features in a patient developed after four cycles of antiPD-1 treatment for lung cancer. The images at the pulmonary trunk depict (**A**) multiple bilateral peribronchovascular consolidations lobe as well as an extensive lower lobe consolidation. A 4-week CT follow-up shows the regression of the previous consolidation but the appearance of new ones (**B**), representing the “migratory” aspect of the organizing pneumonia.

**Figure 3:**
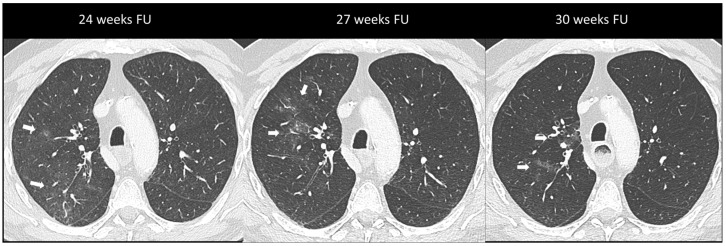
Immuno-related pneumonitis with simple pulmonary eosinophilia pattern pathologically confirmed in a patient with metastatic esophageal cancer receiving chemotherapy and antiPD-1 treatment. The follow-up scans show multiple “migratory” patchy ground-glass opacities in the upper right lobe (arrow).

Imaging plays a major role in the identification, characterization, and follow-up of pulmonary irAEs. Chest X-ray is not recommended due to its limited sensitivity and specificity in detecting ICI pulmonary abnormalities [23]. Chest CT is the preferred imaging modality for suspected ir-pneumonitis with common radiological patterns including organizing pneumonia (OP), simple pulmonary eosinophilia (SPE), hypersensitivity pneumonitis (HP), nonspecific interstitial pneumonia (NSIP), and acute interstitial pneumonitis/diffuse alveolar damage (AIP/DAD) [23,25,39]. Organizing pneumonia is the most frequent pattern and it is generally marked by peribronchovascular or subpleural consolidations or patchy ground-glass opacities, which often migrate over subsequent scans (Figure 2). Interestingly, the “halo sign”, a typical OP feature characterized by ground-glass opacity with a peripheral rim of consolidation, is uncommon in ir-pneumonitis. The extent of the lung involvement correlates with the severity of pneumonitis. A simple pulmonary eosinophilia pattern is usually detected in asymptomatic patients and presents as transient and migratory ground-glass opacities or nodules that may regress spontaneously (Figure 3). Nonspecific interstitial pneumonia typically shows bilateral and symmetrical peripheral ground-glass opacities with lower lobe predominance, which may be associated with reticulations and traction bronchiectasis, while hypersensitivity pneumonitis manifests as centrilobular nodules and lobular hypoattenuations representing small-airway disease. Acute interstitial pneumonitis/diffuse alveolar damage pattern is generally reported in most severe cases and it is characterized by extensive bilateral ground-glass opacities and dependent alveolar consolidations with bronchiectasis appearing in subsequent scans. Micronodules in three-in-bud distribution resembling infectious bronchiolitis as well as a single nodular or mass-like appearance mimicking malignancy have also been described [23,39,40]. Contrast injection is not required; however, it might be performed in routine cancer restaging or in suspected complications such as pulmonary embolism.

Typically, ir-pneumonitis exhibits avidity in ^18^F-fluorodeoxyglucose positron emission tomography-computed tomography (^18^F-FDG PET/CT), which may allow for early detection, sometimes even before symptoms manifest [23]. Nevertheless, FDG uptake does not allow to differentiate it from infection. Accurately characterizing pulmonary abnormalities with ^18^F-FDG PET/CT may be difficult due to free-breathing artifacts, thick slice thickness, and a wide field of view and a complementary CT may be required to better assess features and extent.

**Figure 4:**
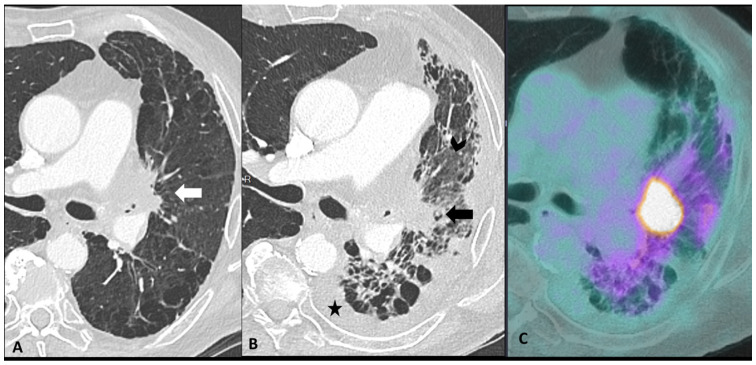
Mimicker of immune-related pneumonitis: tumor progression in a patient with unresectable stage III receiving consolidation immunotherapy after chemoradiotherapy. At baseline CT (**A**) the peri-hilar lesion appearance after radiation therapy is seen (arrow). The restaging CT four months after the introduction of antiPD-1 (**B**) shows the appearance of an extended ground-glass opacity (arrowhead) and septal thickening in the left lower lobe as well as the increase in the size of the peri-hilar mass (arrow) and a semi-circumferential pleural effusion (star), findings suspicious for tumor progression with lymphangitic involvement rather that immune-related or radiation-related pneumonia. The intense uptake of the peri-hilar lesions at PET/CT (**C**) confirms the tumor progression.

Distinguishing between ir-pneumonitis, infection, and tumor progression at imaging may be not straightforward as they often share similar radiological features (Figure 4) [23]. Ir-pneumonitis not only may present as nodular or consolidative lesions mimicking malignancy but it also frequently affects the regions where primary lung cancer and/or lung metastases are located, misleading tumor progression or recurrence [41]. Another challenge in differential diagnosis occurs in case of new-onset lung abnormalities in the radiotherapy field as radiation pneumonitis typically presents with an organizing pneumonia pattern and can manifest even beyond 12 weeks post-radiotherapy due to “radiation recall” phenomena triggered by ICIs [23,41]. Although ir-pneumonitis is primarily located in the radiotherapy field, unlike radiation pneumonitis, it often extends beyond it [41,42]. An additional important challenge for oncologists is identifying the specific drug responsible for pulmonary toxicity in patients receiving ICI in combination with other treatments in particular with molecular targeting therapy such as EGFR TKI, antiangiogenic TKIs, and vascular endothelial growth factor receptor (VEGF) inhibitors [36]. In fact, since they share similar patterns, the identification of the responsible drug is often challenging. The timing of drug introduction and the awareness of the typical features associated with different treatments may aid in identifying the implicated drug and discontinuing it [39,43].

Bronchoscopy with BAL analysis is useful to rule out infection/inflammation and malignancy as well as to assess the cellular pattern by flow cytometric analysis, cytopathology, and microbiological analysis. In the context of ir-pneumonitis, a predominance of lymphocytes with higher than normal CD8+ counts supports the diagnosis, although a mixed pattern is common [44,45]. In cases of simple pulmonary eosinophilia pattern, an increased eosinophil count is found. The additional cytokine and immune cells analysis may support the diagnosis since IL-6, IL-17, and IFN-γ IF are higher in ir-pneumonitis compared to other diseases [46]. Rarely, histological examination may be necessary for a definitive diagnosis [47]. However, despite this crucial role for cytological and pathological analysis, bronchoscopy with BAL or biopsy remains underutilized [38].

Management depends on clinical severity with close monitoring recommended for grade 1 with isolated radiologic changes [10,30]. Grade 2 or higher grade of toxicity warrants ICI discontinuation and treatment with steroids and in severe toxicities, corticosteroid-refractory, or relapse situations, an additional immunosuppressive therapy could be indicated. It is important to note that up to 20% of patients may develop steroid resistance which is associated with poorer outcomes [48]. A peculiar phenomenon called “pneumonitis flare” resulting in pneumonitis recurrence after steroids held without ICI rechallenge has also been described [49]. Rechallenge with ICI after pneumonitis resolution can be attempted in only one-third of patients and requires careful monitoring as recurrent pneumonitis may occur in up to 43% of patients [38,48]. Steroid tapering should be conducted slowly, as relapses of pneumonitis may occur especially in case of organizing pneumonia. Chronic ICI pneumonitis has been reported in 2% of patients characterized by persistent symptoms and radiological abnormalities despite long-term steroids and ICI discontinuation [50].

#### 2.2.2. Sarcoidosis-Like Reaction

Sarcoid-like reactions are side effects generally associated with mono or combotherapy with anti-CTLA-4 (5–7%) [51]. During imaging, the typical sarcoidosis-like pattern appears as symmetrically enlarged mediastinal and hilar lymph nodes often along with perilymphatic micronodules (scissural and peribronchovascular distribution) with variable—from mild to intense—uptake at ^18^F-FDG PET/CT. Since a sarcoidosis-like reaction develops in the first weeks after therapy initiation, attention must be paid before ruling out not only disease progression but also pseudoprogression as it may present as symmetric lymph node enlargement as well [23,51]. Distinguishing these entities may be challenging. Typically, in pseudoprogression, the tumor burden increase is not confirmed at the following imaging assessment [24]. In case of the persistence of further progression in size or FDG uptake, a cytopathologic evaluation is required to distinguish between a sarcoid-like reaction and tumor progression. In a sarcoidosis-like reaction, lymphocytosis with an inverted CD4+/CD8+ ratio and non-caseating granuloma are expected at BAL and on bronchial and/or transbronchial biopsies, respectively [9]. Usually, sarcoidosis-like reactions do not require specific treatment. Treatment holding is required in case of grade > 2 clinical manifestations [10].

### 2.3. Cardiac Adverse Events

Cardiac irAEs, while uncommon, occurring in up to 1% of patients treated in monotherapy and 5.8% in combination therapy, pose a significant threat with high morbidity and mortality rates [52]. The range of cardiovascular complications with ICIs spans from myocarditis to non-inflammatory diseases such as pericarditis, arrhythmias, Takotsubo syndrome, and atherosclerosis-related events. The mechanisms underlying are complex and not yet fully understood, but may involve autoimmune-mediated inflammation, direct cardiotoxic effects, or exacerbation of pre-existing cardiovascular conditions [53,54]. Early recognition of cardiac side effects is crucial to prevent severe impairment and death. Since the majority of cardiac toxicities occur in the first cycles of treatment, the 2022 European Society of Cardiology (ESC) guidelines advise troponin, natriuretic peptides, and ECG at baseline and prior to the initial three cycles of ICI treatment as well as transthoracic echocardiogram at baseline for high-risk individuals [10,55]. Management strategies for cardiac toxicity associated with immunotherapy include prompt discontinuation of the offending agent, initiation of immunosuppressive treatment, support therapy, and close monitoring of cardiac function [10].

#### 2.3.1. Ir-Myocarditis

Myocarditis is the more frequent irAE and usually develops in the first weeks of treatment [54]. It predominantly implicates anti-PD-1 therapy (1%), but combination therapy with anti-CTLA-4 shows a higher prevalence (2.4%) and it is often linked to severe presentations [54,56]. Overlap syndromes accompanying myocarditis with other irAEs such as myositis (25%) and myasthenia gravis (10–11%) are associated with mortality in up to 60% of cases [57]. In these cases, neurological symptoms usually precede the cardiac manifestations.

Studies on the pathogenic mechanism of ir-myocarditis show that CTLA-4 and PD-1 are physiologically involved in preserving immune tolerance and thwarting immune reactions towards cardiac antigens and that animal models lacking CTLA-4 or PD-1 develop severe myocarditis characterized by abundant T-cell infiltration [58]. Similarly, T-cells—predominantly CD8+—and macrophage infiltration are found on pathological specimens of patients affected by ir-myocarditis. The clinical presentation is wide, ranging from general discomfort to chest pain, dyspnea, or syncope and it may be accompanied by troponin elevation, arrhythmias, or heart failure [59]. In case of new-onset symptoms or signs of cardiac disease, the diagnostic workup of patients receiving ICIs should include serous markers, such as troponin, electrocardiogram, echocardiography, and cardiac magnetic resonance (CMR) [55]. Moreover, coronary CT or other investigations may be required to rule out other cardiac injury, including coronary artery disease. Although endomyocardial biopsy is the gold standard in myocarditis, it is reserved only for severely compromised patients since it is an invasive procedure associated with rare but life-threatening complications and tissue subsampling [59].

Thanks to its wide availability, echocardiography is the cornerstone in the assessment of cardiac disease [59,60]. CMR, in addition to standard morphological and functional information by cine imaging, provides robust qualitative and quantitative evaluation of tissue alterations occurring in cardiomyopathies [59]. Myocardial inflammation can be detected as myocardial edema, which is identified by focal or global myocardial hyperintensity in T2, as well as hyperemia and capillary leak as early hyperenhancement on post-gadolinium images [59]. These changes can be also quantitatively measured in T2 and native T1 mapping [59]. Collagen deposition after inflammation leads to a fibrotic scar which is detected as a patchy, subepicardial, and mid-wall late gadolinium enhancement (LGE) in non-vascular territories, with increased values in extracellular volume mapping [59]. The application of the 2018 Lake Louise Criteria which include at least one T1-based criterion (increased myocardial T1 relaxation times, extracellular volume fraction, or LGE) and at least one T2-based criterion (increased myocardial T2 relaxation times, visible myocardial edema, or increased T2 signal intensity ratio) yield high accuracy in detecting myocarditis. According to available studies, myocarditis secondary to ICIs fulfill the Lake Louise Criteria in fewer than half of cases, mainly due to a low incidence of edema detected on T2 in these patients [61,62]. LGE is the most important finding in ir-myocarditis, being reported in 48–80% of patients, frequently affecting the septal wall with patchy mid myocardial or subepicardial distribution [62,63]. Interestingly, mapping values are lower than those reported in myocarditis from other causes [62,63]. Nevertheless, T1 values correlate to major adverse cardiac events [61]. These differences in CMR findings may be which may be due to the prompt administration of steroids in ICI patients presenting cardiac manifestations.

^18^FDG PET/CT has shown high performance in detecting cardiac inflammation; however, it is not routinely used in clinical practice due to its high costs and limited availability [64,65]. The incorporation of ^68^Ga-DOTATOC, a ligand targeting somatostatin receptors, has proven to offer a highly sensitive approach for detecting ir-myocarditis through PET/CT, particularly in recognizing early stages of myocardial inflammation [66]. Additionally, ^68^Ga-DOTATOC PET/CT is valuable in identifying concomitant myositis [66]. For 18F-FDG PET/CT, patients were asked to fast for >6 h before the tracer injection, and blood glucose levels were checked before the tracer injection. Acquisitions were performed 60 min after 18F-FDG injection. For 68Ga-DOTATOC PET/CT, images were obtained 90 min after injection. Emerging molecularly targeted tracers including probes for T-cell detection and status evaluation may represent a promising strategy in the early detection of inflammatory responses [67]. The upregulation of fibroblast activating protein (FAP) is another potential marker of early stages of myocarditis. PET/CT with fibroblast activating protein inhibitor (FAPI) revealed diminished or absent tracer uptake in ICI-treated patients without immunological adverse effects or cardiac impairment [68].

#### 2.3.2. Pericardial Effusion/Pericarditis

Pericardial effusion with or without pericarditis is the second most frequent manifestation of cardiac toxicity, and it can occur alone or associated with myocarditis [65]. Typically minor in quantity, the risk of cardiac tamponade escalates with abundant and circumferential involvement.

#### 2.3.3. Other Ir-Cardiac Adverse Events

**Figure 5:**
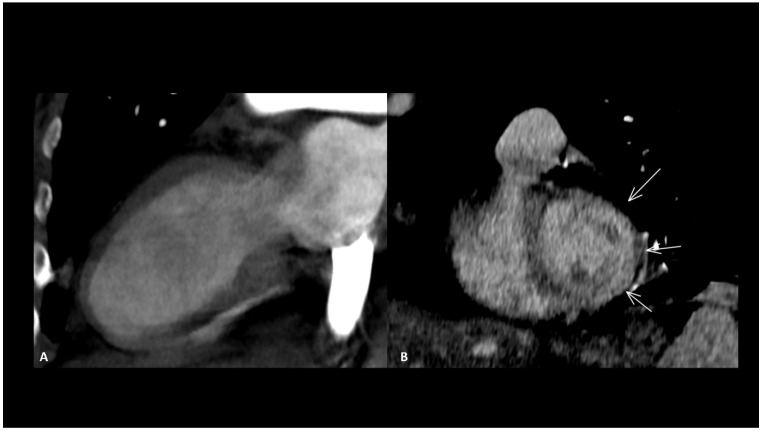
Immune-related Takotsubo disease in a patient treated with anti-PD-1 for recurrent cervical cancer. The patient presented sudden functional impairment with extended akinesia of the mid-cavity and apical wall, with a drop of up to 35% of the ejection fraction. A CT acquired for pulmonary embolism shows apical and mid-cavity ballooning of the left ventricle (**A**) in the two-chamber view and extended myocardial enhancement of the mid-wall (arrows) (**B**). Due to the severe clinical compromise, cardiac magnetic resonance was not performed. The diagnosis was obtained after complete recovery of the ventricle function a few days later.

Other reported cardiac toxicities include non-inflammatory cardiomyopathy with impaired function, arrhythmias, and Takotsubo syndrome, either occurring independently or as a consequence of myocarditis [24,69]. These cardiac manifestations associated with ICIs typically carry a worse prognosis compared to those arising from other causes [70]. Takotsubo disease is characterized by an acute but reversible left ventricular systolic impairment with severe heart failure caused by physical or emotional stress, which typically recovers in a few days or weeks. It generally affects the apical wall in 80% of cases [71]. This localized impairment produces a typically regional ventricular dilatation sparing the basal segment, called “ballooning” which can be seen in the end-diastole (Figure 5). CMR is the modality of choice to assess function, the ballooning feature as well as T2 myocardial edema [71]. Rare cases of coronary spasm after ICI administration which promptly resolved with nitroglycerin have been reported [65]. In the absence of pre-existing coronary heart disease, the pathogenetic mechanism suspected is a T-cell-mediated vasculitis.

### 2.4. Abdominal Adverse Events

#### 2.4.1. Ir-Enterocolitis

IrAEs in the digestive system encompass a range of manifestations. Diarrhea is the most frequent clinical presentation. In a recent systematic review and meta-analysis, the incidence of diarrhea was 12.1–13.7% for anti-PD-1 and 30.2–35.4% for anti-CTLA-4. The incidence of colitis was 0.7–1.6% for anti-PD-1, 5.7–9.1% for anti-CTLA-4%, and 13.6% for the combination of both therapies [72]. Several risk factors of enterocolitis due to immunotherapy have been identified including the type of ICI (combotherapy > anti-CTLA-4 > anti-PD-1/L1), the dose of ICI, and pre-existing inflammatory bowel disease [73]. One study identified elevated levels of interleukin-17, one of the central inflammatory cytokines upregulated in inflammatory bowel disease, in patients with ipilimumab-induced colitis [74]. These findings raise the possibility of using interleukin-17 blockade as a strategy for treating colitis induced by immune checkpoint blockade.

One notable consequence of abdominal irAEs is their role as a primary cause for treatment discontinuation. The median time to the full onset of abdominal irAEs falls within the range of 5 to 10 weeks after initiating immunotherapy. This relatively early manifestation underscores the need for vigilant monitoring during the initial phases of treatment.

Clinical presentation includes increased frequency of bowel movements, loose stools, abdominal cramping, and dehydration. It is essential for healthcare providers to assess the onset, frequency, and duration of diarrhea to differentiate it from other potential causes.

An associated colitis must be suspected in case of high-grade diarrhea, abdominal pain, rectal bleeding, or systemic symptoms such as fever and weight loss. The grading of severity of gastrointestinal (GI) events according to the European Society for Medical Oncology (ESMO) guidelines is reported in Table 3 [10].

Distinguishing immunotherapy-induced colitis from other forms of colitis is critical for appropriate management. Detailed medical history, including the type of immunotherapy, dosage, duration of treatment, and consideration of concomitant medications and any pre-existing gastrointestinal conditions are key elements for the diagnosis. Laboratory tests, including complete blood count, serum electrolyte panel, C-reactive protein (CRP), cytomegalovirus PCR, and stool analysis for enteropathogens and clostridium difficile toxin, aid in assessing the severity of the enterocolitis and to rule out other causes. Elevated inflammatory markers, such as CRP and erythrocyte sedimentation rate (ESR), may indicate underlying inflammation.

Colonoscopy remains the gold standard for diagnosing immunotherapy-induced colitis. Endoscopic findings may include mucosal inflammation, ulcerations, and friability. Biopsy samples obtained during colonoscopy can provide histological confirmation of ir-enterocolitis. However, several possible lesion patterns can be depicted [75]. The most common pattern is focal active colitis, characterized by areas of infiltration with neutrophilic polymorphonuclear cells, cryptitis, and crypt abscesses, which are nonspecific and pose a real problem in terms of differential diagnosis with an infectious origin. Often, the clinical history and microbiological assessment will determine the diagnosis. Another pattern is microscopic colitis, where the mucosa appears endoscopically normal but exhibits a histological appearance of lymphocytic colitis characterized by lymphocytic infiltration of the epithelium, or collagenous colitis associated with subepithelial fibrous thickening. All these patterns are associated with varying degrees of underlying neutrophilic inflammation. Finally, there is the apoptotic pattern, which is suggestive of an immune origin. This pattern, usually observed in graft versus host disease or with immunosuppressive treatments (such as mycophenolate mofetil), is characterized by an increase in apoptotic bodies, and involuted, dilated glands that disappear (vanishing glands).

**Figure 6:**
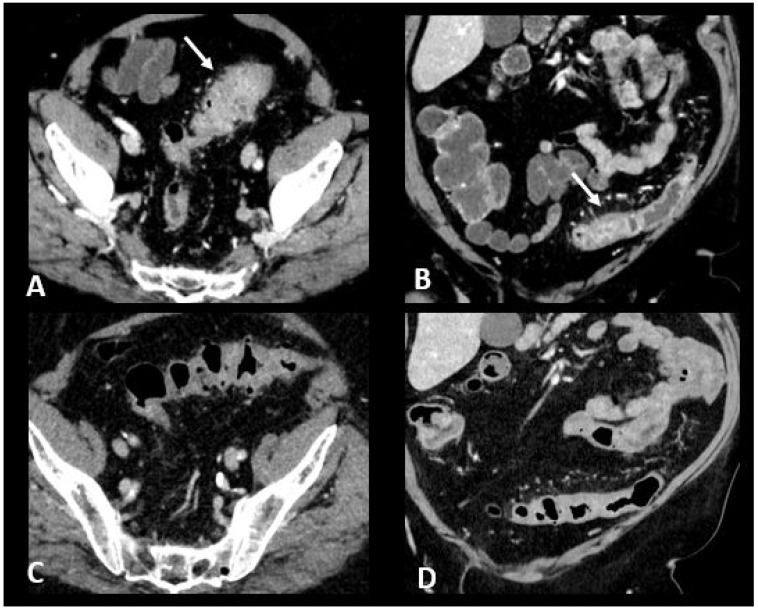
Immune-related colitis in a patient with a lung epidermoid carcinoma treated with nivolumab + ipilumimab. After 2 cycles of immunotherapy, the patient experienced grade 3 diarrhea. Transverse (**A**) and coronal (**B**) CT images show a segmental thickening of the sigmoid mimicking sigmoid diverticulitis (white arrows). The CT images in transverse (**C**) and coronal sections (**D**) performed after treatment cessation show a significant regression in the thickening of the sigmoid wall.

CT is recommended in case of grade 3/4 diarrhea/colitis to assess the extent and severity of colitis. CT protocol includes a single acquisition at the “enteric phase” or “portal venous phase”, meaning around 50 s or 65 s, respectively, after an iodinated contrast agent intravenous injection. Oral contrast agents may be administrated according to the institutional customs. Ir-colitis often exhibits a distinct preference for the distal colon, with the small bowel occasionally affected, typically in conjunction with colitis. A notable imaging feature is the presence of diffuse bowel inflammation combining a bowel wall thickening > 4 mm with mucosal hyperenhancement, congestion of mesenteric vessels, and air-fluid level [24]. Immunotherapy-related colitis may also manifest as segmental colitis. The rectosigmoid region, in particular, tends to be prominently affected, and there is a potential for mimicking symptoms and imaging characteristics of sigmoid diverticulitis. Thickening of the terminal ileum has also been reported [76]. Examples of ir-enterocolitis are shown in Figure 6. PET/CT, when performed for oncologic evaluation, has been reported to be more sensitive than CT scans for early detection of immunotherapy-related entero-colitis which appears as an intestinal heterogeneous, moderate to marked, metabolic activity [24]. However, caution is warranted due to the lack of specificity, especially in patients treated with metformin, where markedly increased non-pathological uptake is frequently observed and can cause complexities in interpretation [77]. Complications such as ischemia, necrosis, hemorrhage, and toxic megacolon can arise as serious and potentially life-threatening consequences during the course of immunotherapy treatment [78]. Severe acute colitis may also result in colonic perforation and death, particularly when there are delays in diagnosis. Previous reports have shown colon perforation in 0.7–1.5% of melanoma patients [79] and 6.6% of renal cell carcinoma patients [80]. In melanoma phase III trials, 0.6–1% of patients died of complications related to ipilimumab-induced enterocolitis [81].

As for other irAEs, management depends on gravity [10]. For grade 1, symptomatic approaches including maintaining adequate hydration and diet, as well as the use of cholestyramine, are recommended. In case of a more severe disease, the treatment should be discontinued, and systemic glucocorticoid administration is mandatory. The way of administration and the dose depends on the gravity and tolerability (i.e., fluids, bland diet). Biologic agents such as infliximab and vedolizumab are used sequentially for patients who do not present a response within 3 days of intravenous glucocorticoid therapy. Although both agents have the same efficacy, vedolizumab is linked to a longer time to achieve clinical response but with a shorter duration of glucocorticoid use [82].

#### 2.4.2. Ir-Pancreatitis

The occurrence of ir-pancreatic toxicity is approximately 4%, and it tends to be more common when anti-PD(L)1–anti-CTLA-4 combination therapy is administered compared to monotherapy [83]. There is limited understanding of ir-pancreatitis, and it is frequently linked with other irAEs, notably enterocolitis (33%) and hepatitis (21%) [83].

It commonly presents asymptomatically, characterized by isolated elevation of amylase and lipase levels. The diagnosis of ir-pancreatitis is made through a process of exclusion which relies on a thorough assessment of medical history, biochemical analyses, imaging, and, if necessary, endosonography with biopsies. Potential differential diagnoses include pancreatic metastases (observed in 13% of patients referred for ir-pancreatitis) and pancreatitis from other causes such as alcohol, hypertriglyceridemia, bile stones or sludge, and drugs other than ICIs.

**Figure 7:**
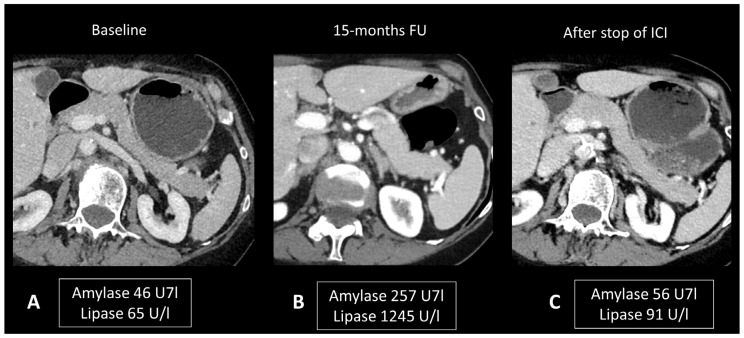
Immune-related pancreatitis in a patient with high-grade serous ovarian carcinoma treated by ipilimumab + nivolumab as the second line of treatment. Transverse CT images performed 15 months after initiation of treatment (**B**), due to elevated lipase and amylase levels, show a subtle diffuse enlargement of the pancreatic gland compared to the baseline CT scan (**A**). Note the regression of this enlargement after cessation of treatment and normalization of pancreatic enzymes (**C**).

Ultrasonography (US) is useful to rule out bile stones. When examined through CT scans, ir-pancreatitis exhibits a pattern resembling pancreatitis from other origins, including focal or diffuse pancreatic enlargement, decreased enhancement, and peripancreatic fat stranding, or a mass-like enlargement such as in autoimmune pancreatitis (Figure 7) [24,83,84]. However, it’s important to note that these Ir-pancreatitis may also present with normal findings. CT or sometimes MRI is recommended as a one-stop-shop examination to assess pancreatitis severity and to rule out pancreatic metastases and other causes of pancreatitis. In particular, by adding cholangiography sequences, MRI can evaluate the presence of pancreas divisum or biliary stones. CT images are acquired at both the “pancreatic phase” and the “portal venous phase”, around 40 and 65 s after iodinated contrast agent injection. MRI protocol includes T2w and T2 Fat Sat for anatomic details and evaluation of necrosis and effusion, and T1w before and after gadolinium agent injection to assess hemorrhage and vascular supply. Post-contrast acquisitions are obtained at the “hepatic phase”, “portal venous phase”, and “interstitial phase”, around 35 s, 65 s, and 3 min after injection, respectively. Cholangiography sequences are three-dimensional heavily T2-weighted sequences that allow the depiction of the biliary and pancreatic ducts by enhancing their signal compared to the surrounding structures. Ir-pancreatitis manifests as increased uptake of ^18^F-FDG in the pancreas on PET/CT scans, with a correlation between the degree of uptake and the severity of pancreatitis [85].

As mentioned above, isolated asymptomatic hyperlipasemia should not prompt any treatment. For symptomatic patients, management includes a clear liquid diet (i.e., bowel rest, intravenous fluids) [10]. The benefits of glucocorticoid administration are not clearly demonstrated and data are limited to retrospective cases [83].

#### 2.4.3. Ir-Hepatitis

Recent studies indicate that 5–10% of patients undergoing ICI monotherapy and 25–30% undergoing ICI-combination therapy experience hepatitis as a secondary effect, with 1–2% and 15% of cases classified as grade 3–4 severity, respectively [10]. Hepatitis secondary to immunotherapy typically manifests within a timeframe of 1 to 15 weeks post-treatment initiation. Hepatitis may exhibit no symptoms, or it can manifest with fever, general discomfort, abdominal pain, jaundice, and loss of appetite. Monitoring liver function through regular biochemical tests, including serum transaminases, alkaline phosphatase (ALP), and bilirubin before each treatment cycle, is imperative during immunotherapy to detect early signs of hepatotoxicity. The diagnosis relies on the results of liver function tests, showing an elevation in alanine aminotransferase (ALAT) and sometimes aspartate aminotransferase (ASAT). To diagnose ir-hepatitis, it is essential to rule out alternative causes of liver injury, such as other medications, alcohol, viruses, metabolic disorders, autoimmune diseases, vascular diseases, and tumor involvement. Interestingly, hepatic metastases and previous systemic or liver-directed therapies have been seen observed to increase the risk of ICI-induced hepatitis [86].

**Figure 8:**
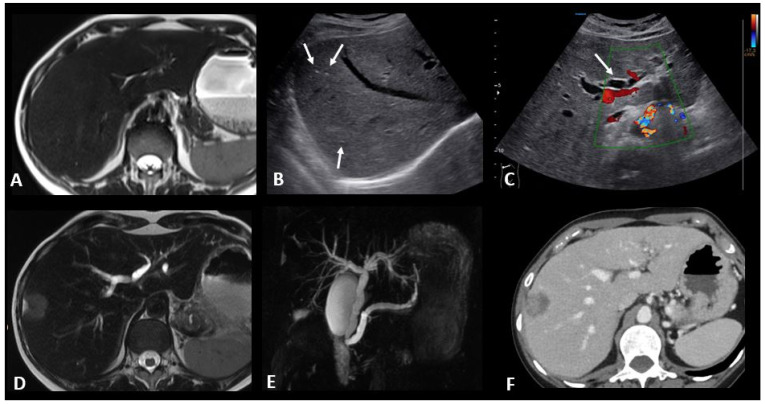
Immune-related hepatitis and cholangitis in a patient with an intestinal-type adenocarcinoma of the duodenum which demonstrated a high tumor burden mutation score (25.4 mut/Mb) and was treated in the first line with ipilimumab + nivolumab. One month after the start of treatment, there was an increase in ASAT, ALAT, and ALP to 2, 3, and 10 times the normal levels, respectively. EBV and CMV PCR in the blood, hepatitis serology, and HIV were negative. The abdominal ultrasound images in B-mode (**B**) and Doppler (**C**) show a heterogeneous liver parenchyma echogenicity with small echogenic foci (starry sky) (arrows figure (**B**)) and the appearance of intrahepatic bile duct dilatation (arrow figure (**C**) compared to the baseline T2 MR images (**A**). MR images with T2 (**D**) and cholangioMR (**E**) sequences demonstrate double bile duct and pancreatic duct dilatation with non-tumoral stenosis of the odi sphincter. Contrast-enhanced CT images acquired 2 months after the stop of immunotherapy (**F**) show a nearly complete regression of the dilation of the intrahepatic bile ducts.

The role of imaging is mainly to rule out other causes of liver dysfunction, in particular hepatic steatosis, biliary diseases, and progression disease. US is commonly employed for this purpose, revealing patterns similar to other causes of hepatitis, such as hepatomegaly, diffusely hypoechogenic liver parenchyma, periportal thickening, and perihilar lymph adenopathies. A starry sky pattern, corresponding with the presence of small echogenic foci representing portal triads and portal venous wall throughout diffusely hypoechogenic liver parenchyma, has also been described [87]. Although non-specific, this finding can be seen in immunotherapy-related hepatitis and often correlates with the severity of hepatitis and the degree of aminotransferase elevation. CT and MRI examinations most often appear normal [76]; however, sometimes they show imaging features of acute liver dysfunction, such as hepatomegaly, heterogeneous parenchymal enhancement, periportal/gallbladder edema, and perihepatic ascites (Figure 8) [24]. In patients with primary cholestasis, MRI with cholangiography is helpful to rule out other liver diseases such as biliary stones and primary sclerosing cholangitis. For MRI protocol, see the “ir-pancreatitis” paragraph.

Contrary to other causes of liver dysfunction, increased liver FDG avidity has been reported on PET/CT in ir-hepatitis. However, the diagnosis is limited by physiological tracer uptake and a reversal of the liver-to-spleen ratio due to higher spleen uptake resulting from ICI-induced T-cell activation.

In terms of pathology, three distinct patterns of histological liver injury induced by ICI have been identified: hepatic (52%), cholangitic (19%), and mixed (29%) [88]. In most cases, inflammatory infiltrates in the portal and lobular regions were lymphocytes, along with the occasional presence of eosinophils, neutrophils, and only sparse plasma cells. Lobular necrosis was also observed in most patients. When comparing ir-hepatitis and other causes of autoimmune hepatitis (AIH), plasma cells were more abundant in inflammatory infiltrates of AIH-patients than in those treated with ICI, whereas centrilobular injury as well as granuloma formation was more prevalent in ir-hepatitis. A trend toward increased CD8+ T-cells was also observed among hepatitic irAEs compared to AIH [88].

Recognition of these complications is paramount, as delayed management may result in secondary complications such as cholecystitis or pancreatitis. Routine monitoring of liver tests before every cycle of ICI is mandatory to early detect hepatobiliary toxicity. Withholding any potential liver toxic medication, including alcohol and alternative medicine and close follow-up of liver tests, is recommended for grade 1. For grades 2 and 3, withholding ICI is mandatory and the rapid initiation of steroids is debated for grade 2; the American Society of Clinical Oncology (ASCO), ESMO, and National Comprehensive Cancer Network (NCCN) guidelines propose close follow-up every 2–3 days and initiation upon persistence or deterioration whereas the society for immunotherapy of cancer (SITC) guidelines recommend the initiation of steroids in any grade 2. For steroid refractory hepatotoxicity, the addition of immunosuppressant therapies is recommended. Mycophenolate mofetil is widely accepted as the first-line standard approach. Upfront azathioprine remains a possible alternative but is less recommended due to the later onset of action. For patients who are refractory to second-line immunosuppressants, tacrolimus (an oral macrolide antibiotic) and tocilizumab (a monoclonal antibody directed against IL6), have shown clinical activity in case reports and small case series. Furthermore, infliximab is not recommended by most guidelines as it is associated with a risk of hepatoxicity.

#### 2.4.4. Ir-Cholangitis

Ir-cholangitis is an uncommon adverse event that can impact either large bile ducts, small ducts, or both. Ir-cholangitis, characterized by inflammation of the bile ducts, can lead to obstructive jaundice and impaired bile flow. Elevated levels of γ-glutamyltransferase (GGT) and ALP are more noticeable than those of transaminases [89].

A recent systematic review analyzed imaging of 29 cases of ir-cholangitis with a large-duct type and 20 with a mixed type [90]. The primary observations included bile duct dilation, stenosis, and irregular thickening of the bile duct wall, which could manifest as segmental or diffuse. Five cases were isolated to the intrahepatic bile duct, fifteen affected the extrahepatic bile duct, and eighteen involved both intra- and extrahepatic bile ducts. Patients with the large-duct type had a higher incidence of abnormalities in the extrahepatic bile duct (53.8% vs. 8.3%) and a lower incidence in the intrahepatic bile duct (3.8% vs. 33.3%) compared to those with mixed cholangitis. Cholangioscopy was performed in five cases, revealing band-like narrowing of the biliary tract wall in three cases, two of which were accompanied by diverticulum-like outpouching. Ulcerative lesions with ‘burned-out’ epithelia were detected in one case. Another case showed multiple scarred lesions with hemorrhage and narrowing of second-order biliary branches. Immune-related cholangitis exhibits a pattern resembling infectious cholecystitis on ultrasound, characterized by gallbladder distension and wall thickening. On MRI, it shares similarities with other causes of cholangitis, presenting as localized or diffuse non-obstructive hypertrophy and hyperenhancement of the extrahepatic bile duct wall, often accompanied by segments of dilatation and narrowing (Figure 8). For MRI protocol, see the “ir-pancreatitis” paragraph.

Liver biopsies reveal evidence of cholangiopathy in most patients. Pathological findings typically included portal inflammation and bile duct injury, and less frequently ductular reaction, bile duct loss, cholestasis, and lobular injury. Biopsies of the extrahepatic bile ducts can show inflammatory infiltration in the lining epithelium and noncurricular diffuse fibrosis as characteristic associated findings [90].

As for hepatitis, ir-cholangitis management requires laboratory test monitoring for 2–3 days before the introduction of steroids according to ASCO, ESMO, and NCCN guidelines [10].

### 2.5. Ir-Endocrinopathies

Ir-endocrinopathies are common immunotherapy toxicities, mainly including hypothyroidism, hyperthyroidism, hypophysitis, primary adrenal insufficiency (PAI), and insulin-dependent diabetes mellitus. Treatment discontinuation and steroids may be not required in most cases, while the alteration often becomes chronic, thus requiring hormone replacement.

#### 2.5.1. Ir-Hypophysitis

**Figure 9:**
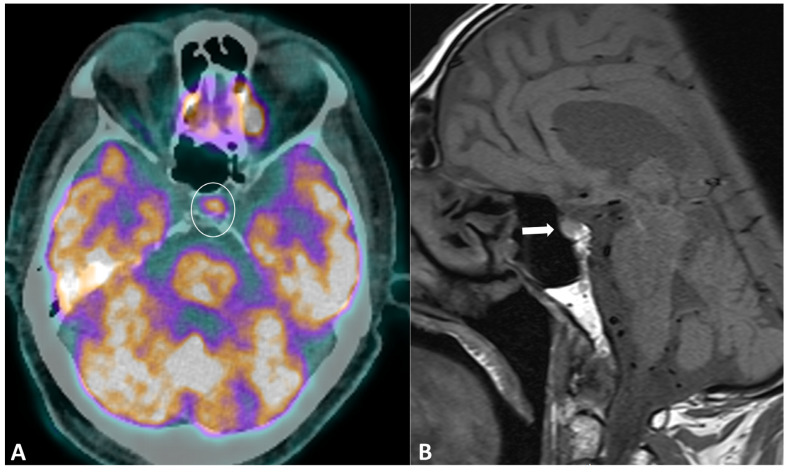
Immune-related hypophysitis in a patient with metastatic lung cancer treated with combined antiPD-1 + antiCTLA-4. A restaging PET/CT (**A**) showed the appearance of a moderate uptake of the hypophysis (circle). An MRI was then performed, revealing (**B**) a slight hypophyseal enlargement with homogeneous enhancement (arrow), without significative pituitary stalk thickening noroptic chiasm compression. The hormonal tests were unremarkable, and thus compatible with a grade-1 hypophysitis. ICI treatment was temporally discontinued to ensure the regression of the metabolic activity and the absence of delayed clinical or biological signs, then rechallenged with no recurrence of the hypophysitis.

Hypophysitis is an ICI complication, occurring in 0.1% to 18% of patients, depending on the type of treatment [91]. The clinical presentation is nonspecific, with symptoms derived from decreased cortisol (fatigue, appetite loss, nausea, vomiting, malaise, dizziness, and mild cognitive defects) being far more common than in primary hypophysitis [92]. The diagnostic workup usually includes hormonal axes evaluation and hypophysis MRI. Pituitary biopsy is not necessary, it is rarely performed in case of alternative diagnosis (metastasis) suspicion. MRI protocol includes T1w and T2w sequences with small FOV in coronal and sagittal planes for assessing size and anatomical details, followed by dynamic T1w acquisitions after gadolinium contrast agent injection at multiple time points using small FOV. MRI can be normal in up to 30% of cases [92]. When pathological, the key finding is a diffuse and transient enlargement of the pituitary gland (Figure 9). The enhancement pattern can be variable, either homogenous [93] or geographic with a reduced enhancement of the antehypophysis [94]. The pituitary stalk can also be thickened. The differential diagnosis includes pituitary metastasis and adenoma. The most reliable elements for the differential diagnosis are the regression of the enlargement after treatment interruption and, for metastasis, the absence of invasion and destruction of neighboring structures. Typically, ir-hypohysitis shows intense FDG uptake at PET/CT and its detection may precede symptoms (Figure 9).

#### 2.5.2. Ir-Thyroiditis

Thyroid toxicities are commonly observed, with reports of up to 10% of patients in clinical trials although rates tend to be higher in real-world settings [95]. Occurrences are rarer with anti-CTLA4 compared to combination therapy and anti-PD-1 [10,96]. The underlying mechanism involves a T-cell-mediated autoimmune response, cytokines, and autoantibodies targeting thyroglobulin thyroid peroxidase and thyroid stimulating hormone (TSH) receptor, resulting in inflammation followed by destructive thyroiditis [97]. The axis antiPD-1 and anti-PDL-1 also play a crucial role, as their involvement is already known in autoimmune thyroiditis and Graves’ disease. By blocking the PD-1/PD-L1 axis, ICIs reduce protection against immune-mediated destruction [98].

Evidence suggests that ICIs may trigger latent thyroiditis, as indicated by elevated baseline TSH levels, as well as positivity for TPO or Tg antibodies in patients developing ir-thyroiditis [95,99]. Therefore, pre-existing thyroid disease before initiating ICI treatment should be routinely assessed [97]. Thyroid dysfunction may be asymptomatic or present with thyrotoxicosis followed by hypothyroidism or with hypothyroid manifestations. It predominantly affects women and can occur at any time during therapy, with a peak incidence observed 6–10 weeks after treatment initiation, often resolving spontaneously in most cases [100]. Isolated hypothyroidism is more commonly associated with anti-CTLA-4, while thyrotoxicosis is frequently observed with anti-PD-1 and anti-PD-L1 inhibitors. Discontinuation of ICIs is generally not required, as most cases are subclinical or low-grade [10].

**Figure 10:**
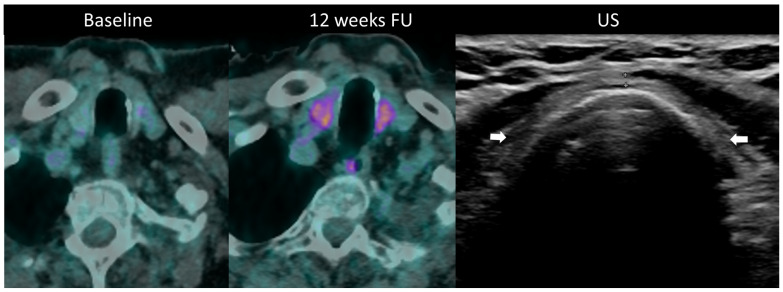
Immune-related thyroiditis in a patient receiving chemotherapy and combined anti-PD-1 + anti-CTLA-4 for metastatic lung cancer. At baseline 18F-FDG PET/CT, no thyroid uptake is seen. At week 16, a diffuse moderate metabolic activity of the thyroid gland appeared at PET/CT. The patient developed a grade 2 primary hyperthyroidism. After the resolution of the acute phase, the laboratory tests showed a marked primary hypothyroidism and the ultrasound depicted an atrophic and heterogeneously hypoechogenic thyroid gland (arrows) consistent with a pre-existing chronic thyroiditis.

Diagnosis is established through laboratory monitoring of TSH and free T4 every 4–6 weeks [97]. T3 measurement is warranted in hyperthyroidism. Although imaging is not routinely used for diagnosing thyroid disorders during ICI therapy, morphological and metabolic abnormalities are often visible. Ultrasound may be utilized to assess temporal changes such as gland enlargement and heterogeneous echogenicity in the acute phase, as well as the development of hypotrophy in persistent hypothyroidism; hypervascularity on color Doppler imaging is seen in thyrotoxicosis [24]. Diffuse hypoattenuation is often observed on CT scans, accompanied by changes in gland size, and increase in thyrotoxicosis, and a decrease in hypothyroidism [101]. ^18^F-FDG PET/CT typically reveals increased gland uptake (Figure 10). Importantly, it has been noted that baseline PET/CT demonstrating thyroid hypermetabolic activity is associated with an increased risk of developing ir-thyroiditis.

#### 2.5.3. Ir-Adrenalitis

**Figure 11:**
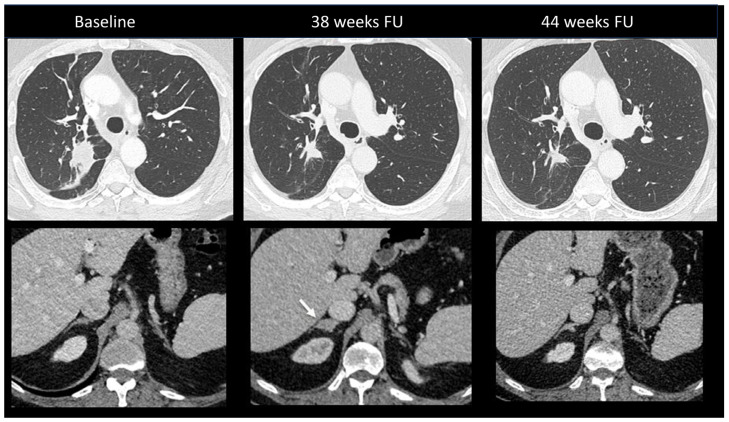
Immune-related adrenalitis in a patient with a lung carcinoma treated with Nivolumab. CT images taken 38 weeks after the start of treatment demonstrate a significant decrease in the size of the primary lung tumor compared to baseline, but an increase in size with a nodular appearance of the right adrenal gland suspected for a new metastasis (arrow). Despite this finding, immunotherapy was continued. Subsequent CT images performed 44 weeks after treatment revealed the disappearance of the enlargement of the right adrenal gland, confirming the diagnosis of immune-related adrenalitis.

Primary adrenal insufficiency is a rare but severe complication occurring in up to 5% of patients treated with combination ICI [10]. Onset is insidious, with non-specific symptoms ranging from asthenia and nausea to typical adrenal insufficiency characteristics. No specific features have been reported at imaging, with no abnormalities detected in most cases [102]. Rarely, gland enlargement or atrophy as well as increased FDG uptake may be observed [24,103]. Figure 11 shows an ir-adrenalitis characterized by symmetrical enlargement.

### 2.6. Ir-Rheumatological and Musculoskeletal Adverse Events

**Figure 12:**
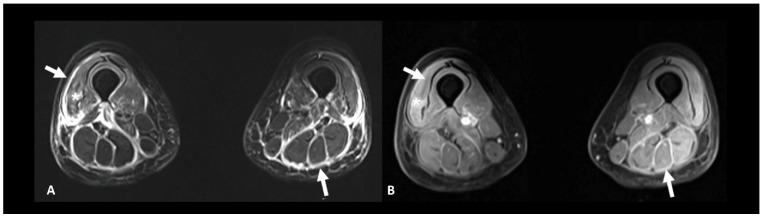
Immune-related myositis/fasciitis in a patient with a history of glioblastoma, under antiPD-1. T2 (**A**) and post-contrast T1 fat-suppressed (**B**) images show muscle edema with enhancement (asterisks), as well as thickening and enhancement of muscular fascia (arrows) distributed in the anterior and posterior compartments of both thighs. Lesions regressed under steroid therapy.

Rheumatic and musculoskeletal toxicities are reported in up to 10% of patients [10]. Myositis, with or without fasciitis, is a rare but severe complication that needs to be ruled out in case of myalgia. MRI is useful to confirm the diagnosis by showing muscle edema (Figure 12). Inflammatory arthritis, including polymyalgia rheumatica, can occur in 5–10% of patients [10]. Ultrasound is the modality of choice to depict synovial hypertrophy, tenosynovitis, articular effusion as well as synovial hyperemia on power Doppler analysis; however, MRI provides a more thorough and less operator-dependent assessment of the inflammatory activity by showing soft tissue and bone marrow edema as well as synovial enhancement [104]. Typically, increased uptake of the articular surfaces is seen at ^18^F FDG PET/CT, but articular erosions are rarely found in this context.

### 2.7. Ir-Dermatological Adverse Events

Cutaneous adverse events are the most common ICI toxicities, affecting more than one-half of patients, and usually occur within the first 6 weeks after treatment initiation [10]. The clinical appearance is wide, they are often accompanied by pruritus. Since they are usually mild events, treatment holding is not required. The lesions may be seen during restaging imaging, appearing as non-specific subcutaneous or cutaneous nodules, with mild to intense uptake at PET/CT.

## 3. Adverse Events Related to CAR-T Cell Therapy

CAR-T cells, which are genetically engineered immune cells, may also develop adverse events, including cytokine release syndrome (CRS), neurotoxicity including immune effector cell-associated neurotoxicity syndrome (ICANS), hematologic toxicities leading to cytopenia, hypogammaglobulinemia, tumor lysis syndrome, dermatologic, cardiovascular, pulmonary and gastrointestinal toxicities, infusion-related reactions and infections [105]. The frequency and severity mainly depend on disease burden, CAR-T cell proliferation, and the host inflammatory condition.

### 3.1. Immune Effector Cell-Associated Neurotoxicity Syndrome

ICANS is the most common neurological immunotherapy adverse event, touching 23–67% and 40–62% of patients with lymphoma and leukemia, respectively, with severe events reported in 12–30% and 13–42% of patients with lymphoma and leukemia, respectively [106]. Clinical presentation is wide, including encephalopathy/delirium, aphasia, depressed level of consciousness, seizure, headache, tremor/myoclonus, motor dysfunction, dysarthria, neuropathy, meningismus, hemiparesis, and hallucinations [107]. The diagnostic workup usually includes lumbar puncture, contrast-enhanced brain MRI, and EEG. Because coagulopathy can occur with severe ICANS, serologic testing for coagulopathy may also be included.

**Figure 13:**
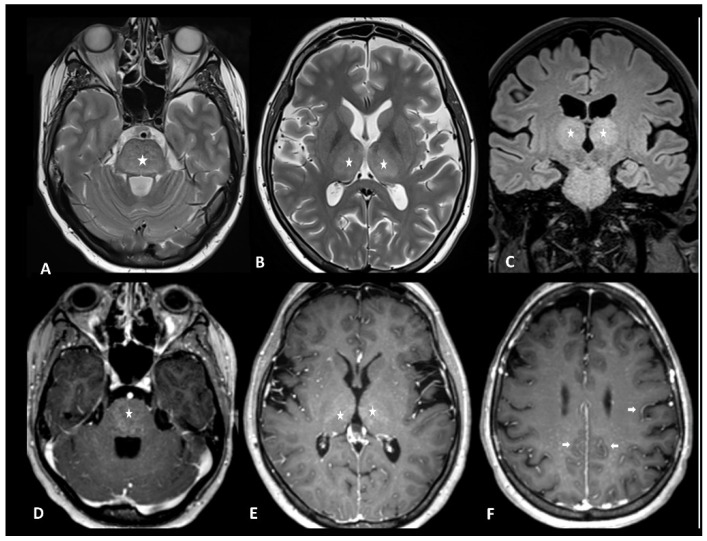
Immune effector cell-associated neurotoxicity syndrome in a 28-year-old female patient suffering from diffuse large B-cell lymphoma. The patient was admitted for CAR T infusion in the context of disease progression. Twenty-four hours after the first infusion, the patient developed fever, tachycardia, dysphagia, and dyskinesia. MRI revealed bithalamic and brain stem hyperintensities (asterisks) on T2 and FLAIR (**A**–**C**) and contrast enhancement (**D**,**E**). Diffused perivascular spaces enhancement (arrows) was also observed (**F**). No sign of infection was found during biological tests. Despite intravenous methylprednisolone infusion, the patient died 5 days later.

Neuroimaging is unremarkable in most cases but can show T2 and FLAIR hyperintensity (especially bilateral thalami and brainstem), vasogenic edema, leptomeningeal enhancement, multifocal microhemorrhages, cortical diffusion restriction, and transient corpus callosum lesions [108] (Figure 13). Diagnostic workup with radiological clues and main differential diagnoses are reported in Table 4.

### 3.2. Hemophagocytic Lymphohistiocytosis/Macrophage Activation Syndrome

Hemophagocytic Lymphohistiocytosis/Macrophage Activation Syndrome (HLH/MAS) is a rare but potentially fatal complication of CAR T-cells, touching 1.0–3.5% of patients undergoing CAR T-cell therapy [109]. It is a systemic disorder and presents with neurological abnormalities in one-third of cases.

Diagnostic workup includes chest x-ray, brain MRI, blood sampling, and bone marrow biopsy. During MRI, classical findings are cerebral atrophy, cerebral edema, hemorrhage, calcification, necrosis, leptomeningeal enhancement, and ventriculomegaly with prominent extra-axial fluid [110]. Table 3 shows a diagnostic workup with radiological clues and main differential diagnoses.

### 3.3. Cytokine Release Syndrome (CRS)

CRS is the most frequent toxicity, occurring in 37–93% and 77–93% of patients with lymphoma and leukemia, respectively, with severe events reported in 1–23% and 23–46% of patients with lymphoma and leukemia, respectively [106]. It is marked by an excessive immune response, and it is characterized by fever and other, nonspecific, manifestations like malaise, myalgia, gastrointestinal issues, tachycardia, and rash. It appears within the first hours to 4–7 days after administration and it is generally self-limited. Half of patients require intensive care hospitalization [106].

Imaging is generally not required for CRS diagnosis [111]. Pleural effusion with atelectasis and pulmonary edema are common complications of CAR-T-cell therapy occurring during CRS which can be detected at CT [105,111]. Rare cases of other pulmonary adverse events have been reported. These include organizing pneumonia patterns, such as for ICIs (see Section 2.2.1) and CAR-T-cell infiltration [112,113,114]. CAR-T-cell infiltration manifests as new-onset consolidations, nodules, reticulations, and peripheric ground-glass opacities mimicking superimposed infection or lymphoma recurrence, as the latest CT findings are peribronchovascular consolidations or nodules [114]. In Table 5 the different CT patterns of CAR-T cells thoracic complications are described along with the main clues for the identification and the main differential diagnoses. Cardiac complications have been reported in up to 10% of patients, ranging from arrhythmia, cardiomyopathy, myocardial infarction, and heart failure [115]. Diagnostic work-up is similar to that for ICIs, including serum markers, EEG, echocardiography, and, in selected cases, CMR and angioCT or coronarography to assess coronary arteries (see Section 2.3).

## 4. Conclusions

Immune-related adverse events represent an important complication that may arise during immunotherapy, affecting both treatment efficacy and patient well-being. The spectrum of the toxicities consequent to immunotherapy is wide and depends on the type of treatment and the organ target.

A thorough understanding of the clinical and radiological features of immunotherapy toxicities and their differential diagnosis is crucial for accurate diagnosis and effective treatment, thereby preventing diagnostic delays, minimizing treatment disruption, and optimizing the benefits of immunotherapy. A multidisciplinary collaboration is necessary for early identification and management for patients’ benefit. Future research aimed at identifying risk factors and pathogenetic mechanisms could potentially prevent these events. Meanwhile, advancements in machine learning may enable the early detection of these toxicities at a preclinical stage.

## Figures and Tables

**Table 1:** Neurotoxicities related to the Immune Checkpoint Inhibitors, diagnostic work-up with main diagnostic and non-radiological clues, and main differential diagnoses.

Toxic Effect		Diagnostic Work−Up	Main Differential Diagnosis	Main Radiological Clues
***CNS***	Encephalitis	CE brain MRI + LP + EEG + autoantibodies	Infectious encephalitis, disease progression	Mesiotemporal T2/FLAIR hyperintensities
	Aseptic meningitis	CE brain MRI + LP	Infectious meningitis, disease progression	Leptomeningeal enhancement
	CNS vasculitis	Angio−CT or MRI + LP + serum vasculitides markers +/− biopsy	Stroke for other etiologies, radiation vasculopathy, disease progression, infectious disease	Microangiopathy and subarachnoid hemorrhage on SWI, cortical and subcortical restricted diffusion
	Demyelinating disease	CE brain and spine MRI + LP + antibodies +/− CE optic nerve MRI	Multiple sclerosis, neuromyelitis optica spectrum disorder, transverse myelitis, isolated optic neuritis	Cortical, juxtacortical, periventricular, or infratentorial T2/FLAIR hyperintensities +/− enhancement
	Hypophysitis	CE brain MRI	Pituitary metastasis, adenoma	Diffuse and transient enlargement of the pituitary gland
	Transverse myelitis	CE spine MRI + LP + autoantibodies	multiple sclerosis, neuromyelitis optica spectrum disorder, infectious myelitis	T2 hyperintensities > vertebrae, patchy contrast enhancement
***PNS***	Neuropathy	Serologic testing+ NCS/EMG +/− CE spine MRI	Infectious neuropathy, Guilla Barré syndrome, leptomeningeal carcinomatosis	Nerve root enhancement
	Neuromuscular junction disorders	Myasthenia antibody evaluation, EMG, pulmonary function testing	/	/
	Myopathy	Serum-specific antibodies, screening for concurrent myocarditis, EMG +/− muscle biopsy	Other causes of myopathy	Muscular T2 hyperintensities

CNS: central nervous system; PNS: peripheral nervous system; CE: contrast-enhanced; EEG: electroencephalogram; LP: lumbar punction; MRI: magnetic resonance imaging; NCS/EMG: nerve conduction studies/electromyography; Readapted from: Burton LB, Neuro-Oncol Adv. 2021; Albarrán V. Front Pharmacol. 2022. [11,15].

**Table 2:** Radiological patterns, main clues, and differential diagnoses of thoracic toxicities by immune checkpoint inhibitors.

Radiological Pattern		Main Radiological Findings	Main Radiological Clues and Tips	Main Differential Diagnosis
***Pneumonitis***	OP	Multifocal, patchy, consolidations or GGOs with peribronchovascular and/or subpleural distribution	Migratory on a subsequent scan	Radiation Pneumonitis Infectious pneumonia Tumor progression
	SPE	Scattered nodules or GGOs	Migratory and transient	Infectious Pneumonia
	HP	Bilateral and symmetric GGOs or poorly-defined centrilobular micronodules with upper lobes predominance and hypo-attenuated lobules	Expiratory acquisition to confirm the air-trapping in case of doubt	Infectious pneumonia (mainly viral and pneumocystis) Respiratory bronchiolitis
	NSIP	Bilateral and symmetric areas of GGOs +/− irregular reticulations, with peripheral and/or peribronchovascular distribution and lower lobes predominance. Bronchiolectasis and bronchiectasis often absent	Prone positioning acquisition to confirm the abnormalities in case of doubt	Infectious interstitial pneumonia (mainly viral and pneumocystis) Poor ventilation of dependent lung areas HP
	AIP/DAD	Bilateral dependent consolidation associated with GGO	Absence of reticulations and peribronchovascular thickening related to cardiac edema	Infectious pneumonia Cardiogenic oedema
	Bronchiolitis	Centrilobular nodules in tree-in-bud distribution	/	Infectious bronchiolitis
	Solitary Nodule	Nodule(s)	Mild metabolic activity at 18F-FDG PET/CT	Lung cancer Metastasis Infectious pneumonia including septic emboli
***Other toxicities***	Sarcoidosis-like Reaction	Enlarged hilar and mediastinal lymph nodes +/− bilateral perylimphatic (mainly scissural) micronodules	Bilateral and symmetrical	Lymph node metastasis disease Lymphangitis
	Pleural Effusion	Bilateral, small entity	Absence of nodular pleural thickening and pleural enhancement Absence of metabolic activity at 18F-FDG PET/CT	Malignant pleural effusion Pleural carcinosis

OP: organizing pneumonia; SPE: simple pulmonary eosinophilia; HP: pulmonary hypersensitivity; NSIP: non-specific interstitial pneumonia; AIP/DAD: acute interstitial pneumonitis/diffuse alveolar damage; GGOs: ground glass opacities.

**Table 3:** Grade of GI adverse events according to the ESMO guidelines.

Grade	Mild G1	Moderate G2	Severe G3	Life-Threatening G4
Symptom	Increase of <4 liquid stools/day	Increase of 4–6 liquid stools/day	Increase of ≥7 liquid stools/day	Any grade and one of the following: hematochezia, abdominal pain, mucus in stool, dehydration, fever

ESMO guidelines [10].

**Table 4:** Neurotoxicities related to the CAR-T-cell therapy, diagnostic workup with radiological clues, and main differential diagnoses.

Toxic Effect		Diagnostic Workup	Main Differential Diagnosis	Main Radiological Clues
***CNS***	ICAN	LP + CE brain MRI + EEG	Toxic leukoencephalopathy	Bilateral thalami and brainstem T2/FLAIR hyperintensity
	Hemophagocytic Lymphohistiocytosis	brain MRI + blood sampling + bone marrow biopsy	/	Periventricular white-matter abnormalities, brain-volume loss, enlargement of extra-axial fluid spaces

CNS: central nervous system; CE: contrast-enhanced; EEG: electroencephalogram; LP: lumbar punction; MRI: magnetic resonance imaging;: nerve conduction studies/electromyography.

**Table 5:** The radiological patterns, main clues, and differential diagnosis of thoracic toxicities by CAR-T-cell therapy.

Radiological Pattern		Main Radiological Findings	Main Radiological Clues and Tips	Main Differential Diagnosis
***CRS***	Pleural effusion	Bilateral and symmetrical pleural effusion and passive atelectasis	Gravitational distribution; absence of enhancement	Disease progression
	Oedema	Peribronchial thickening and reticulations may be associated.	The normal size of pulmonary veins	Cardiac edema
	OP	Multifocal, patchy, consolidations or GGOs with peribronchovascular and/or subpleural distribution	Migratory on a subsequent scan	Infectious pneumonia Disease progression
	CAR-T cells infiltration	Consolidation with peripheral ground-glass opacities, nodules, reticulations	Temporal relation with therapy infusion	Infectious pneumonia Disease progression

CRS: cytokine release syndrome; OP: organizing pneumonia.

## Abbreviations

ALAT	alanine aminotransferase
ASAT	aspartate aminotransferase
ASCO	American Society of Clinical Oncology
BAL	bronchoalveolar lavage
CAR-T cell	chimeric antigen receptor T-cell
CIP	checkpoint-induced pneumonitis
CMR	cardiac magnetic resonance
CNS	central nervous system
CRP	C reactive protein
CT	computed tomography
CTCAE	common terminology criteria for adverse events
CTLA-4	cytotoxic T-lymphocyte antigen-4
EGFR	epidermal growth factor receptor
EEG	electroencephalogram
EMSO	European Society for Medical Oncology
FAPI	fibroblast activation protein inhibitor
FLAIR	fluid attenuated inversion recovery
GI	gastrointestinal
GGT	gamma-glutamyl transferase
HLH/MAS	hemophagocytic lymphohistiocytosis/macrophage activation syndrome
ICI-P	ICI-pneumonitis
ICIs	immune checkpoint inhibitors
ICANS	immune effector cell-associated neurotoxicity syndrome
IR	immune-related
LGE	late gadolinium enhancement
LP	lumbar puncture
MRI	magnetic resonance imaging
NCCN	National Comprehensive Cancer Network
NSIP	nonspecific interstitial pneumonia
OP	organizing pneumonia
PAI	primary adrenal insufficiency
PD-1	programmed death-1
PD-L1	programmed death-ligand 1
SITC	Society for Immunotherapy of Cancer
SPE	simple pulmonary eosinophilia
TKIs	tyrosine kinase inhibitors
TSH	thyroid stimulating hormone
US	ultrasound
VEGF	vascular endothelial growth factor
